# Long-term efficacy of lenvatinib for recurrent papillary thyroid carcinoma after multimodal treatment and management of complications: a case report

**DOI:** 10.1186/s12885-018-4612-2

**Published:** 2018-06-28

**Authors:** Masayuki Tori, Toshirou Shimo

**Affiliations:** Department of Endocrine Surgery, Osaka Police Hospital, Kitayamacho 10-31, Tnnoujiku, Osaka, 543-0035 Japan

**Keywords:** Lenvatinib, Multimodal treatment, Papillary thyroid cancer, Tyrosine kinase inhibitor, Tracheal perforation

## Abstract

**Background:**

The advent of tyrosine kinase inhibitors (TKIs) has changed the treatment of RAI refractory, unresectable recurrent differentiated thyroid cancer (DTC), which was formerly treated with multidisciplinary remedies.

**Case presentation:**

Here we describe the case of a 64-year-old woman who underwent total thyroidectomy with tracheal resection and suffered from a recurrent tumor in the neck and multiple lung and bone metastases 3 and 11 months, respectively, after the operation. Multimodal therapies, RI (I-131), EBRT, and taxane-based chemotherapy were ineffective, and sorafenib was started as a TKI. However, because of disease progression, sorafenib was replaced by lenvatinib after 9 months. The effect of lenvatinib has continued for more than 1 year and 9 months, and the patient has well survived. During the treatment period, a tracheal pin-hole fistula suddenly emerged, which was naturally cured by the temporary cessation of lenvatinib. Adverse events such as hypertension, proteinuria, and diabetes as innate complications have been successfully managed until the present according to our institute regulations.

**Conclusions:**

Even where multimodal treatment was ineffective, lenvatinib was suggested to be an alternative treatment option for RAI refractory recurrent DTC and patient could have a chance to be controlled successfully.

## Background

Generally, differentiated thyroid carcinoma (DTC) has good prognosis, and the standard treatment of locally advanced DTC is surgery with occasional radioactive iodine therapy. In contrast, patients who develop recurrence or metastatic radioactive iodine refractory disease have a 10-year survival rate of only 15–20% [1, 2]. Before the advent of tyrosine kinase inhibitors (TKIs), the rarely effective chemotherapy was the only available remedy for RAI refractory DTC; sometimes, external beam radiotherapy (EBRT) [3] and volume reduction surgery were included in optional therapies. In 2014, sorafenib, a multi-target kinase inhibitor (m-TKI), became available in Japan after it was found to be effective in the phase 3 DECISION study [4]; lenvatinib was then approved according to the results of the phase 3 SELECT study. Sorafenib is an m-TKI that targets VEGFR 1–3, RET, RAF, and PDGF-β, whereas lenvatinib targets VEGFR 1–3, FGFR 1–4, RET, KIT, and PDGF-α [5]. For the use of m-TKIs, the definition of RAI and the application and timing of use are important because both of these m-TKIs have various adverse events that lead to dose interruptions and reductions [6]. Because AEs, hypertension, hand–foot syndrome, eruption, proteinuria, diarrhea, fatigue, and hepatic dysfunction are very common, close attention should be paid, particularly to hand–foot syndrome for sorafenib [7] and to hypertension for lenvatinib [8]. In addition, care needs to be taken regarding aerodigestive and gastrointestinal fistulas, which, although rarely observed in the phase 3 SELECT study, may become fatal [9]. In this report, we experienced a locally advanced DTC patient who had extended surgery with tracheal resection and rapid growth of recurrent and metastatic tumors after the operation and for whom lenvatinib was proved to be remarkably effective after several other ineffective multidisciplinary remedies.

## Case presentation

**Fig. 1:**
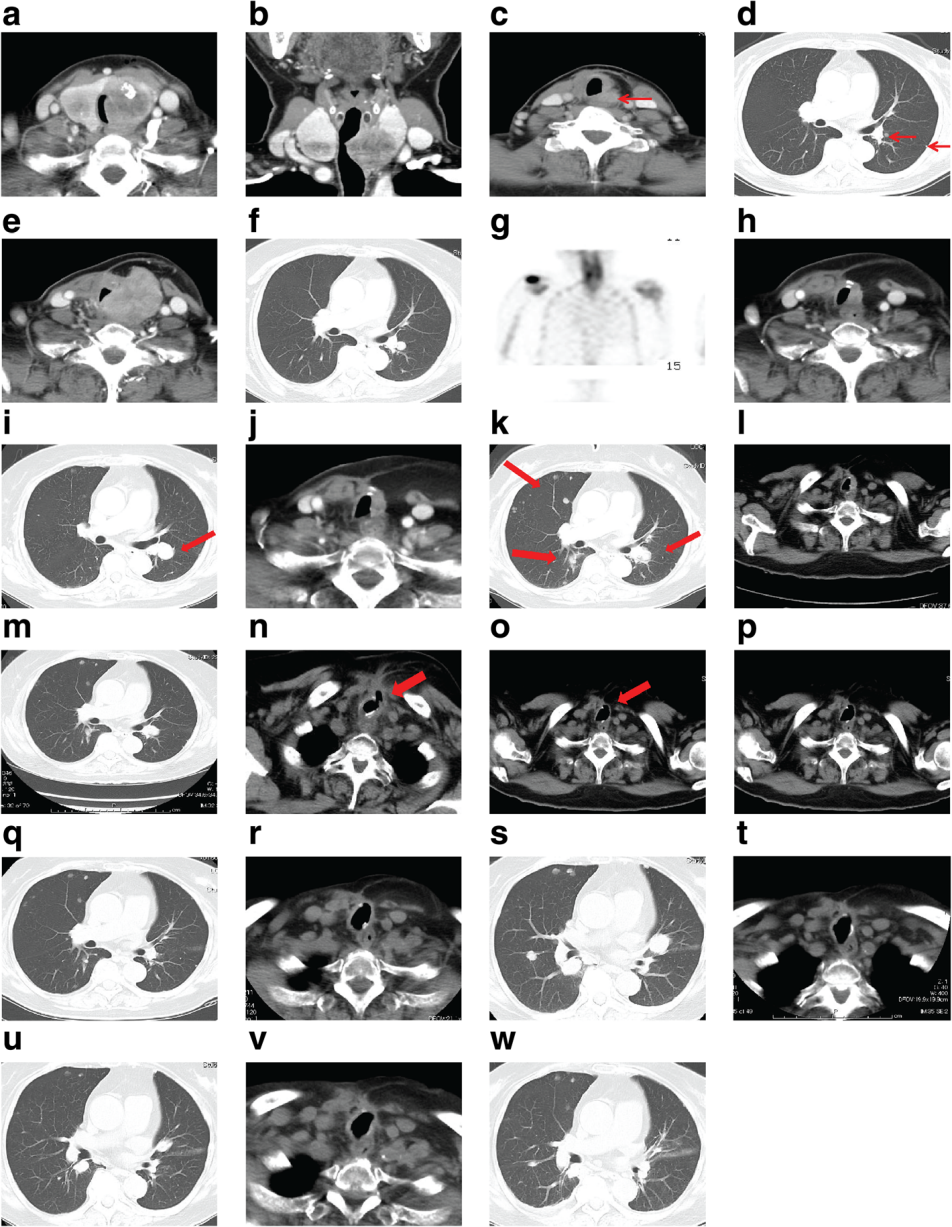
**a** and **b** Enhanced CT findings before operation. The tumor (arrow) was mainly located in the left lobe and invaded into half the tracheal circumference (40 × 36 mm) (Fig.2(A)). **c** and **d** Recurrent tumor was found just below the left piriform fossa (15 × 11 mm) (**c**), and lung metastasis (max, 6 mm) (**d**) was found at the same time 3 months after the operation by CT scan (Fig.2(B)). **e**, **f**, and **g** Nine months after the operation, CT scan showed prominent tumor progression in the neck (48 × 38 mm) (**e**) and the lung (18 × 16 mm) (**f**). Additionally, bone scintigraphy demonstrated a solitary bone metastasis in the right scapula (**g**) (Fig.2(C)). **h** and **i** Three years after the operation, local recurrence in the neck was controlled (15 × 11 mm) (**h**), although multiple metastasis in the lung worsened (PD). Maximum size was 30 × 26 mm (**i**) (Fig.2(D)). **j** and **k** Within 8 months after starting sorafenib, local recurrence (**j**) and lung metastasis worsened (PD) (**k**) (Fig.2(E)). **l** and **m** Within 1 month after starting lenvatinib, the tumor in the neck remained controlled (**l**) and multiple metastases in the lung decreased and diminished in size (18 × 15 mm) (**m**) (PR) (Fig.2(F)). **n** Within 3 months after starting lenvatinib, pin-hole perforation (5 mm) of the trachea suddenly appeared at the end of tracheal invasion (Fig.2(G)). **o** Within 1 month after terminating lenvatinib, the perforation was naturally cured and pin-hole closed (Fig.2(H)). **p** and **q** Within 2 months after restarting lenvatinib, tumors in the neck (**p**) and the lung (**q**) were controlled (PR) (Fig.2(I)). **r** and **s**Although local recurrence (**r**) was kept controlled, lung metastasis (**s**) was exacerbated (21 × 18 mm) for 2 months after terminating lenvatinib because of adverse events (PD) (Fig.2(J)). **t**and **u**Within 3 months after restarting lenvatinib, local recurrence in the neck (**t**) and lung metastasis (**u**) remained under control (PR) (Fig.2(K)). **v** and **w** One year and 9 months after starting lenvatinib, CT scan still showed PR (Fig.2(L))

**Fig. 2:**
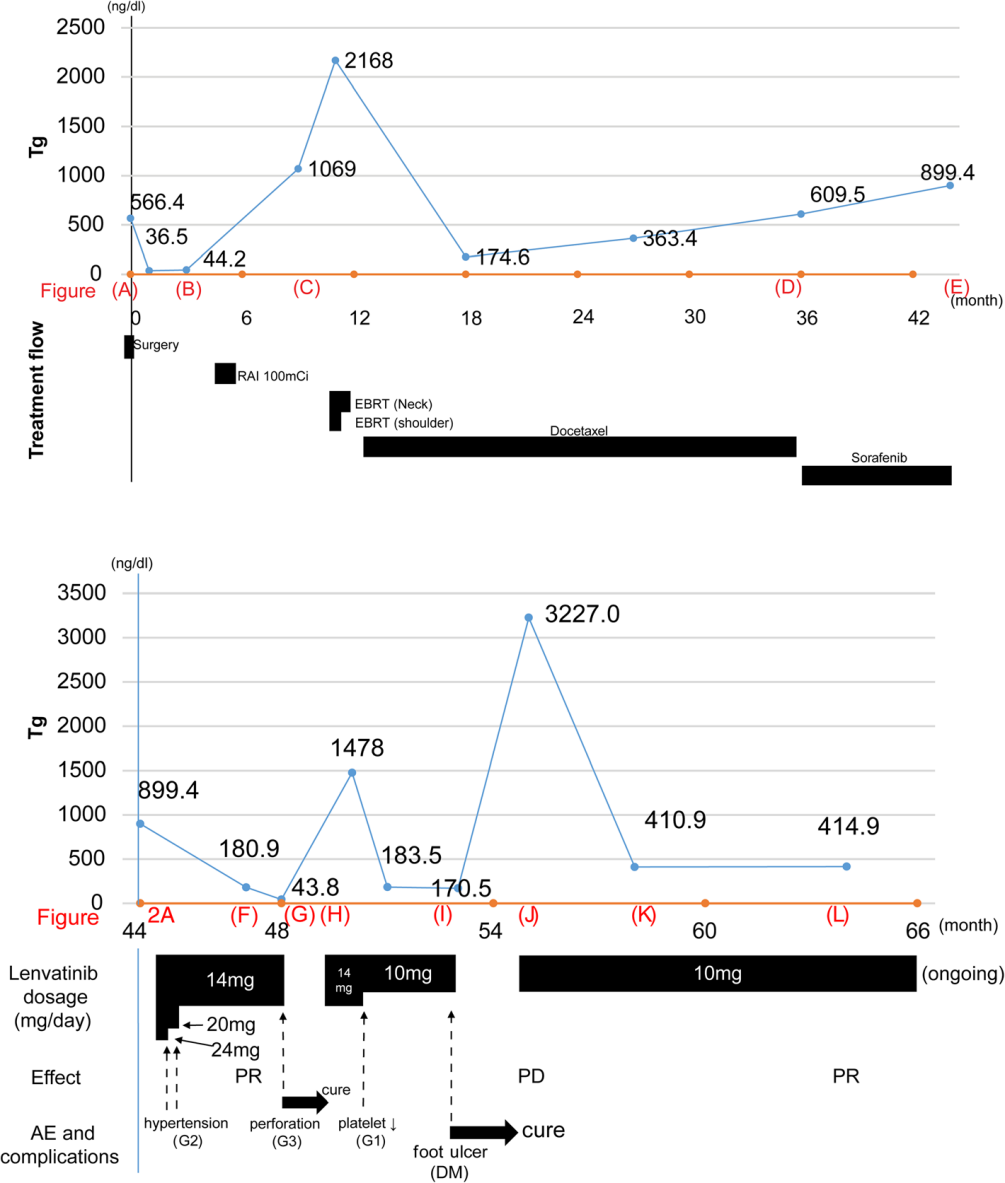
Time-course result of patient remedy and effect, including thyroglobulin level. Alphabets in the figures (shown in red, such as (A)) correspond to the alphabets in Fig. 1

A 64-year-old woman was diagnosed with locally advanced DTC with invasion to the trachea, esophagus, and left recurrent nerve (Fig. 1a, b and Fig. 2(A)). Bronchoscopy revealed that the invasion to the trachea was under half the tracheal circumference, and the distance from the vocal cord to the oral end of the tumor, invasive to the mucosa of the trachea, was 3 cm. Her past medical history included non-insulin dependent diabetes mellitus controlled using insulin injections for a year. She underwent total thyroidectomy with bilateral modified radical neck dissection, followed by a window resection of the trachea invaded by the tumor. A one-stage reconstruction was then performed using an auricular deltopectoral flap. The patient was finally diagnosed with papillary thyroid carcinoma (PTC), pT4aN1bM0, stage IVA, according to the 7th edition of the Union for international cancer control TNM classification of malignant tumors. The operation was macroscopically curative, although a final histopathological estimation of the tracheal margin was positive. Three months after the operation, apart from tracheal anastomosis and the newly emerged lung metastasis, a recurrent tumor was detected outside the left piriform fossa (Figs. 1c, d and 2(B)). Therefore, the patient was given 100 mCi of I-131 therapy. No accumulation of I-131 was detected. Nine months after the operation, the patient felt apparent dyspnea and a dull pain in the right shoulder. A CT scan revealed prominent tumor progression in both the neck and the lung, and bone scintigraphy showed bone metastasis in the right scapula (Figs. 1e, f, g and 2(C)). EBRT was performed for the recurrent neck tumor (60 Gy) and the right scapula (36 Gy), and docetaxel was administered once per 3 weeks for 24 months. Docetaxel was temporarily very effective for the local recurrence, although the lung metastasis was remarkably enlarged (Figs. 1h, i and 2(D)). Three years after the operation, the patient was started with the newly emerged TKI sorafenib, but because of the progression of lung metastasis, it was terminated in 9 months (Figs. 1j, k and 2(E)), although bone scintigraphy demonstrated the disappearance of bone metastasis. Therefore, 45 months after the operation, lenvatinib was started. There are strict regulations regarding the use of lenvatinib at our facility, which must be adhered to (Table 1). Within 2 months after the start of lenvatinib, recurrent tumor and lung metastasis was remarkably decreased [partial response (PR), Figs. 1l, m and 2(F)], but 1 month later, coughing and dyspnea appeared and XP demonstrated pneumonia. A CT scan demonstrated a pin-hole perforation of the trachea (Figs. 1n and 2(G)). The symptoms disappeared 1 month after lenvatinib was terminated, and the tracheal fistula naturally closed (Figs. 1o and 2(H)). Lenvatinib was then restarted, following which the local recurrence decreased and most metastatic tumors in the lung disappeared within 3 months (Figs. 1p, q and 2(I)). However, because of the exacerbation of diabetes involving a foot ulcer, the administration was again halted for 2 months, which led to the exacerbation of lung metastasis (Figs. 1r, s and 2(J)). After restarting the administration, diabetes, hypertension, and urinary protein as adverse events were well controlled by drugs and nutrition counseling and lung metastasis was controlled; CT scan demonstrated no recurrence in the neck, and bone scintigraphy revealed no bone metastasis (Figs. 1t, u and  2(K)). Till the present, lenvatinib has continued to be effective (PR) 1 year and 9 months after the initiation of the drug (Figs. 1v, w and 2(L)). Time-course result of patient remedy and effect, including thyroglobulin level, is shown in Fig. 2.

**Table 1:** Management points for lenvatinib

Items	Contents
Start of lenvatinib	Lenvatinib is started under hospitalization (2–3 weeks). Pharmacists guide patients with respect to taking medication. Patients learn regarding adverse events and accompanying symptoms.
Food and nutrition	Meal nourishment instructions are properly provided to reduce adverse events, such as hypertension or proteinuria.
Collaboration with Specialists	During adverse events, patients consult relevant specialists.
Blood pressure (BP)	BP is measured four times per day to estimate the intraday fluctuations. BP control is fundamentally performed according to the guidelines for the management of hypertension 2014 (The Japanese Society of Hypertension). Angiotensin II receptor blockers and calcium channel blockers are recommended.

## Discussion and conclusions

The principle treatment of locally advanced DTC is surgery achieving R0 (no residual cancer) [10, 11]. In this case also, undoubtedly, the initial therapy should be surgery, including tracheal resection and reconstruction. After the operation, aggressive local recurrence and distant metastasis emerged, and as a standard treatment for such a case, a single dose of 100 mCi of radioactive iodine therapy was given with a negative diagnostic scan; therefore, instead of further radioactive iodine treatment, EBRT was primarily indicated for analgesic purpose, and combined systemic chemotherapy was followed. Because a lack of RAI uptake confers a poor prognosis, EBRT and systemic chemotherapy may be effective options. In our case, docetaxel was administered because its effectiveness was formerly suggested [12], and in fact, it was effective for a year; it controlled local recurrence in the neck, but lung metastasis was remarkably worsened. At that time, there was no further line of treatment because TKIs were unavailable; hence, terminal care may have been recommended.

Sorafenib then became commercially available. However, because of disease progression, it was discontinued. Lenvatinib then became available and was taken as the next line of treatment. The phase III SELECT study documented a significant improvement in the median PFS among patients treated with lenvatinib compared with those treated with the placebo (18.3 months vs. 3.6 months; HR, 0.21; 99% CI, 0.14–0.31; *p* < 0.001). Compared with other TKIs, lenvatinib is very effective because it has potency with regard to the inhibition of FGFR1–4, offering a potential opportunity to block a mechanism of resistance to the VEGF/VEGFR inhibitors.

Lenvatinib also has a direct oncogenic effect on the control of tumor cell proliferation by inhibiting RET and an effect on the tumor microenvironment by blocking FGFR [13, 14]. The SELECT study included prior anti-VEGF TKI treatments (sorafenib, 77%; sunitinib, 9%; pazopanib, 5%; and other, 9%), differing from the DECISION study, and these treatments were effective [5]. In fact, we had no data regarding molecular-biological approaches, including western blotting analysis of pathologic specimens or new generation sequencing data of this patient. Nevertheless, we selected lenvatinib as an effective drug because, as Tahara et al. [15] reported, there should be no difference in drug efficacy because of genetic background, and lenvatinib was the last TKI available instead of sorafenib. In this case, lenvatinib was effective after the failure of sorafenib. To our knowledge, this is the first report of metastatic, RAI refractory, unresectable recurrent DTC in which possible multimodal treatments and other TKIs were ineffective, although a report existed indicating the effectiveness of lenvatinib as a fourth-line TKI for thyroid cancer [16].

For an effective use of TKIs, our institute regulations are reported to be very important and effective in introducing TKIs. Because of our adherence to these regulations, the use of lenvatinib has been maintained since a long time and has caused tumor shrinkage.

In contrast, lenvatinib caused tracheal fistula formation close to anastomosis. A history of EBRT is thought to increase the risk of fistulas [9]. In our case, the fistula promptly closed after the cessation of lenvatinib, although there are some reports regarding the delayed healing of fistulas caused by lenvatinib, which is active against the FGFR family of tyrosine kinase receptors [17]. The delayed healing of tissue may have been reversed by the cessation of lenvatinib [18].

Because the patient suffered from diabetic nephropathy, the control of diabetes apart from nutritional remedies—required the discontinuation of lenvatinib, which caused tumor recurrence. Therefore, it is suggested that in such patients, TKI does not kill malignant cells, but just stabilizes them, and that TKI should be given until tumor recurrence.

The timing of the use of TKI should be considered only when the benefits outweigh the risks, but as we cannot usually predict adverse events, patients’ requests might also be important [6]. Although, it should be also emphasized that during the course of the treatment, lenvatinib was very effective, despite two instances of medicine cancellation and its subsequent resumption, which indicates that the effect can be restored by intermittently using the medicine, even if discontinued.

Possible future treatment strategies after the failure of lenvatinib are discussed below. First, lenvatinib and sorafenib are the only TKIs available in Japan; therefore, the already decreased dose of lenvatinib should be increased to certify effectiveness because Morelli and Puxeddu [16] have previously reported that increase in lenvatinib dose enabled disease control. Second, because efficacy of some BRAF or MEK inhibitors have been reported [19], they may also be available in the near future. Finally, immunotherapy is another option: Nivolumab plus Ipilimumab (NCT03246958) or WT1 [20] vaccine may be promising.

To our knowledge, this is the first case report of recurrent PTC for which possible multimodal treatments were finally ineffective (PD) because of disease aggressiveness and the promising TKI lenvatinib was extremely effective (PR) for a long time (more than 1 year and 9 months). Moreover, it should also be noted that this case, which presented the perforation of the trachea invaded by the tumor, could be cured by a temporary cessation of the drug, and thereafter drug intake could be maintained by a proper management of adverse events or complications.

## Abbreviations

DTC: Differentiated thyroid cancer
EBRT: External beam radiotherapy
m-TKI: multi-target kinase inhibitor
PTC: Papillary thyroid carcinoma
TKI: Tyrosine kinase inhibitors
